# Marine stepping‐stones: Connectivity of *Mytilus edulis* populations between offshore energy installations

**DOI:** 10.1111/mec.15364

**Published:** 2020-02-11

**Authors:** Joop W. P. Coolen, Arjen R. Boon, Richard Crooijmans, Hilde van Pelt, Frank Kleissen, Daan Gerla, Jan Beermann, Silvana N. R. Birchenough, Leontine E. Becking, Pieternella C. Luttikhuizen

**Affiliations:** ^1^ Wageningen Marine Research Den Helder The Netherlands; ^2^ Aquatic Ecology and Water Quality Management Group Wageningen University Wageningen The Netherlands; ^3^ Deltares, Marine and Coastal Systems Delft The Netherlands; ^4^ Animal Breeding and Genomics Centre Wageningen University Wageningen The Netherlands; ^5^ Department of Functional Ecology Alfred Wegener Institute Helmholtz Centre for Polar and Marine Research Bremerhaven Germany; ^6^ Helmholtz Institute for Functional Marine Biodiversity Oldenburg Germany; ^7^ Centre for Environment, Fisheries & Aquaculture Science Suffolk UK; ^8^ Marine Animal Ecology Group Wageningen University Wageningen The Netherlands; ^9^ Department of Coastal Systems NIOZ Royal Netherlands Institute for Sea Research Utrecht University Den Burg The Netherlands

**Keywords:** connectivity, *Mytilus edulis*, North Sea, offshore installations, particle tracking models, stepping‐stones

## Abstract

Recent papers have suggested that epifaunal organisms use artificial structures as stepping‐stones to spread to areas that are too distant to reach in a single generation. With thousands of artificial structures present in the North Sea, we test the hypothesis that these structures are connected by water currents and act as an interconnected reef. Population genetic structure of the blue mussel, *Mytilus edulis*, was expected to follow a pattern predicted by a particle tracking model (PTM). Correlation between population genetic differentiation, based on microsatellite markers, and particle exchange was tested. Specimens of *M. edulis* were found at each location, although the PTM indicated that locations >85 km offshore were isolated from coastal subpopulations. The fixation coefficient *F*_ST_ correlated with the number of arrivals in the PTM. However, the number of effective migrants per generation as inferred from coalescent simulations did not show a strong correlation with the arriving particles. Isolation by distance analysis showed no increase in isolation with increasing distance and we did not find clear structure among the populations. The marine stepping‐stone effect is obviously important for the distribution of *M. edulis* in the North Sea and it may influence ecologically comparable species in a similar way. In the absence of artificial shallow hard substrates, *M. edulis* would be unlikely to survive in offshore North Sea waters.

## 1 INTRODUCTION

The past decades have witnessed a steady advance of artificial structures into the marine environment, commonly referred to as “ocean sprawl” (Bishop et al., 2017; Firth et al., 2016). These artificial hard substrates include nearshore elements such as jetties, dikes and beach groins, as well as offshore structures such as oil and gas platforms, wind turbines and buoys. Growth of hard‐substrate flora and fauna on them is typically abundant (Coolen et al., 2018; De Mesel, Kerckhof, Norro, Rumes, & Degraer, 2015; Krone, Gutow, Joschko, & Schröder, 2013). An increase in connectivity for these species is one of the many kinds of effects these structures are expected to have on natural ecosystems (Adams, Miller, Aleynik, & Burrows, 2014). Artificial hard substrates can act as stepping‐stones; that is, patches of natural habitat that are normally unconnected may now be connected through migration in two or more generations (Fowler et al., 2019). Marine stepping‐stones can be important for the distribution of native species such as *Caryophyllia smithii* (Coolen, Lengkeek, et al., 2015), *Porifera* sp. (van der Molen et al., 2018) and *Desmophyllum pertusum* (Henry et al., 2018) and most species present on offshore artificial reefs in the North Sea are indigenous for the area (Coolen et al., 2018; De Mesel et al., 2015). However, the stepping‐stone effect might also contribute to a faster distribution of nonindigenous species (Adams et al., 2014; IPIECA, 2010; Macreadie, Fowler, & Booth, 2011).

A recent review of priority questions that need to be addressed in the field of decommissioning of offshore structures in relation to marine ecology emphasized the need to study the stepping‐stone effect (Fowler et al., 2019). The prediction of increased connectivity due to the presence of a network of artificial hard substrates has so far only rarely been tested directly using genetic markers. A study conducted on the limpet *Patella caerulea* around Italy found significant population structure for microsatellite markers (Fauvelot, Bertozzi, Costantini, Airoldi, & Abbiati, 2009). Significant population structure, and hence only low connectivity, among artificial hard substrates has also been observed in several coral species in the Gulf of Mexico (Atchison, Sammarco, & Brazeau, 2008; Sammarco, Brazeau, McKoin, & Strychar, 2017). In contrast, Sammarco, Brazeau, and Sinclair (2012) showed low levels of differentiation between coral species on artificial structures in the Gulf of Mexico. Furthermore, the polychaete *Pomatoceros triqueter* showed no genetic differentiation among populations from 11 offshore gas platforms in the Adriatic Sea, suggesting that connectivity might be very high in this case (Fauvelot, Costantini, Virgilio, & Abbiati, 2012). In general, sample acquisition from offshore energy installations is highly challenging, especially in the North Sea, as scientists need to build good relationships with several offshore companies. To work on offshore energy installations, scientists require permissions from the respective installation operators, diving companies and vessel companies, manifesting in various contracts. Sample collectors need to hold commercial diving and offshore safety certifications, usually being on standby for months in a row to be available for last minute planned operations and then, when finally offshore, to be allowed to enter the water to acquire samples.

An alternative approach to study connectivity among artificial hard substrates is biophysical modelling of oceanographic currents, tracking the fate of particles in calculated simulations (Hufnagl et al., 2016). For example, the dispersal range of the bryozoan *Watersipora subtorquata* was modelled to include some platforms in the Santa Barbara Channel while other installations were out of reach for the species (Simons et al., 2016). Connectivity between coastal populations of moon jellyfish *Aurelia* spp. increased due to the introduction of platforms in the Adriatic Sea (Vodopivec, Peliz, & Malej, 2017). Using artificial species representing a native and non‐native species, Adams et al. (2014) showed that in two generations, larvae were able to disperse between Scotland and Northern Ireland due to the introduction of artificial structures at dispersal boundaries.

The combined use of both approaches (particle tracking and genetic markers) is considered as the best available practice and was applied to a wide range of taxa in natural marine habitats (Selkoe et al., 2016). While some studies found a congruence between the biophysical and genetic approaches (Buonomo et al., 2017; Hernawan et al., 2017; Schiavina, Marino, Zane, & Melià, 2014; Schunter et al., 2011; Sjöqvist, Godhe, Jonsson, Sundqvist, & Kremp, 2015), other investigations demonstrated discrepancies (Galindo et al., 2010). Furthermore, a congruence may be detected only at larger spatial scales but not at smaller scales (Foster et al., 2012; Johansson et al., 2015) or vice versa (Jorde et al., 2015).

**Figure 1:**
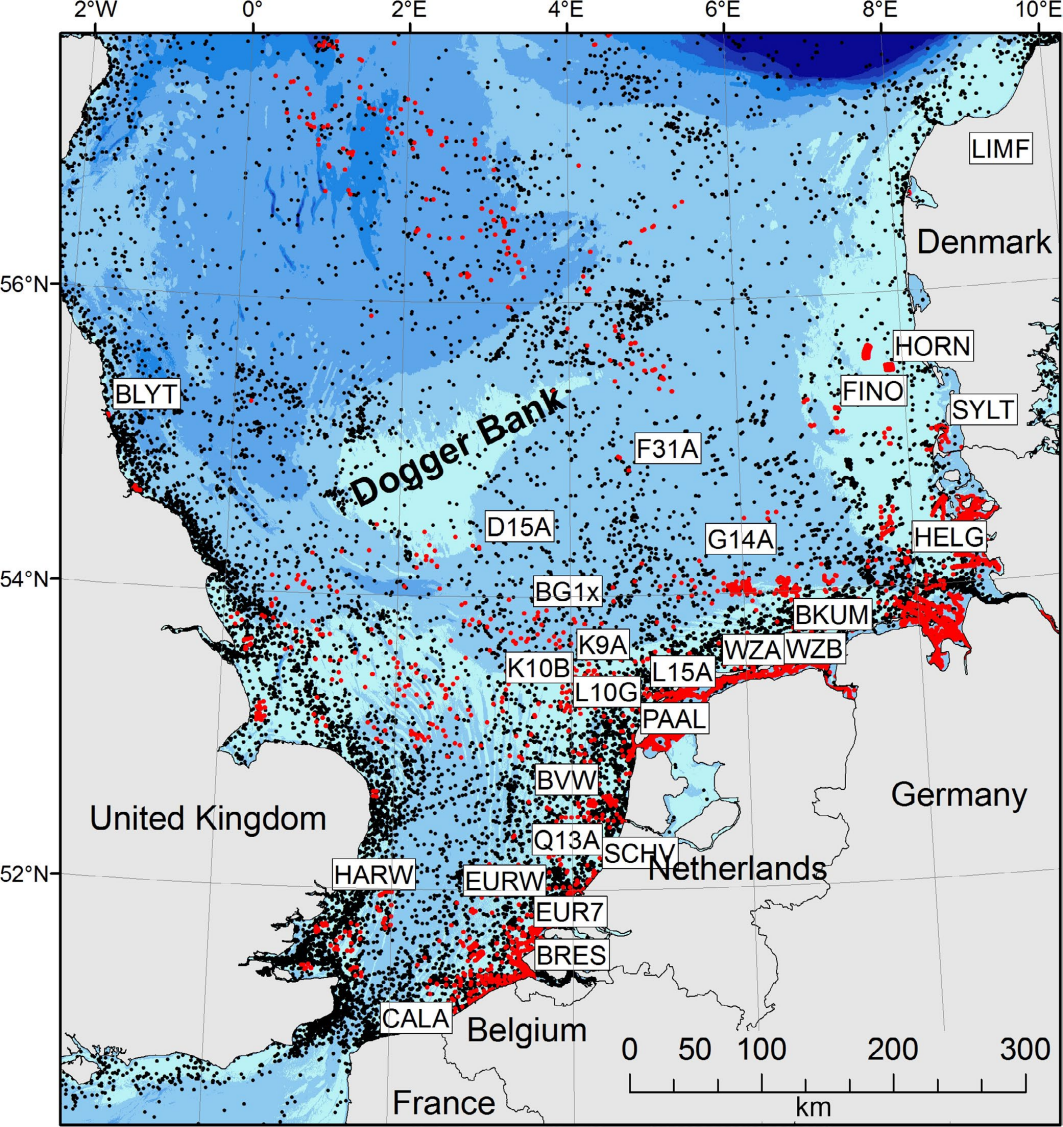
Map of the North Sea displaying all artificial reefs and sample locations considered in this study. Southern North Sea, sampling locations (white labels), artificial structures with surface contact (platforms, wind farms, buoys; red dots) and structures without surface contact (subsea platforms, shipwrecks; black dots). Abbreviations of sample locations are given in Table 1. Note that buoys are only shown for Belgian, Dutch and German waters [Colour figure can be viewed at http://www.wileyonlinelibrary.com/]

Abundant communities of plant and animal taxa grow on offshore artificial hard substrates in the North Sea. Here, we chose to study blue mussels, *Mytilus edulis*, as they are intertidal to shallow subtidal and thus fully rely on artificial substrates offshore; in the North Sea, they do not occur on sea floor habitats such as permanently submerged rocks or gravel (Coolen et al., 2018). *Mytilus edulis* occurs naturally along shorelines of the northeast Atlantic from the high Arctic to the English Channel (Skibinski, Beardmore, & Cross, 1983). It hybridizes in stretched out mosaic hybrid zones with *Mytilus trossulus*, which inhabits the Baltic Sea in the east and with *Mytilus galloprovincialis* in the south (Fraïsse, Roux, Welch, & Bierne, 2014; Hilbish et al., 2012). Populations along the North Sea shores are *M. edulis* (Bierne et al., 2003; Luttikhuizen, Koolhaas, Bol, & Piersma, 2002) and therefore we expected the same for the offshore North Sea. *Mytilus edulis* has a pelagic larval stage of 16–70 days after which the larvae metamorphose to the pediveliger stage during which it is capable of settling on suitable substrates (Bayne, 1965; Filgueira, Brown, Comeau, & Grant, 2014; van der Molen et al., 2018). In the southern North Sea, highest concentrations of *M. edulis* larvae are observed between March and July (P. Kamermans, Wageningen Marine Research, personal communication). Coolen, Bos, et al. (2015) suggested that species with a pelagic larval stage of up to 10 weeks are unlikely to colonize far offshore objects in the North Sea without a source subpopulation located on an upstream intertidal object. However, many platforms and other shallow objects such as wind farms and navigational buoys exist in the North Sea (Coolen et al., 2016; Figure 1), making it likely that coastal larvae reach a suitable artificial substrate at the time the pediveliger stage begins, after which its offspring may be able to reach the far offshore object.

Here, we present results from particle tracking models (PTMs) and validate them using multilocus genetic data for *M. edulis* for the southern North Sea region, an area with abundant offshore human activity. We hypothesized that anthropogenic offshore installations enable *M. edulis* to extend its distribution to areas that would typically not be accessible for coastal populations of this species by the means of a stepping‐stone effect. To our knowledge, this is the first study to combine these two approaches to address connectivity among artificial offshore structures.

## 2 MATERIAL AND METHODS

### 2.1 Study area

The North Sea is located in the North‐East Atlantic and is largely enclosed by land. Tidal water enters the North Sea in the south through the English Channel and from the north between Scotland and Norway. This results in a residual anticlockwise circulation. The North Sea bottom consists largely of sandy and muddy sediments, interrupted by areas with coarse substrates such as gravel and rocks. Many anthropogenic structures provide artificial hard substrates in the North Sea. There are >27,000 shipwrecks, 1,397 offshore oil and gas production installations, ~3,500 wind turbines and a large (but not exactly known to us) number of navigational buoys (Coolen et al., 2016; Wind Europe, 2017).

### 2.2 *Mytilus edulis* population genetics

Samples were collected at 27 locations (hereafter subpopulations) between April 2014 and January 2016: from coastal subpopulations during low tide, by commercial and scientific divers from oil and gas platforms and wind farm foundations, and during inspection, repair and maintenance work on buoys and offshore installations from structures lifted out of the water (Table 1; Figure 1). Sampling depth varied between 0 and 27 m. Two probable outgroup subpopulations corresponding to peripheral populations of the species *Mytilus edulis* (Denmark) and *M. galloprovincialis* (Portugal) were included, from a harbour in Lisbon, Portugal, and from mussel longlines in the Limfjorden in Denmark. Geographical distances between subpopulations (excluding outgroups) varied from 17 km (Scheveningen and Q13‐A) to 1,105 km (Blyth and Sylt) with an average of 348 km.

**Table 1:** Sampling details

Name	Full name	Date	Structure	Size	Depth (m)	Lat.	Lon.	Distance (km)
BG1x	BG 1	11 March 2015	Buoy	44	0	53.8833	3.4983	116
BKUM	Borkum Riffgat	29 June 2014	Wind farm	25	4	53.6900	6.4800	14
BLYT	Blythe	14 November 2014	Pier	48	0	55.1258	−1.4983	0
BRES	Breskens	20 August 2014	Breakwater	48	0	51.4068	3.5121	0
BVW	BV W	30 September 2014	Buoy	48	0	52.6007	3.5170	74
CALA	Calais	20 August 2014	Pier	24	0	50.9661	1.8433	0
D15A	D15‐A	3 October 2015	O&G platform	48	7	54.3247	2.9346	181
EUR7	EURO 7	15 September 2014	Buoy	48	0	51.9900	3.5031	32
EURW	Euro W	24 July 2014	Buoy	48	0	51.9095	2.7232	70
F31A	F3‐1A	1 September 2014	O&G platform	47	5	54.8520	4.6949	166
FINO	FINO 3	23 September 2015	Research platform	28	4	55.1950	7.1583	71
G14A	G14‐A	16 July 2014	O&G platform	48	13	54.2241	5.4986	85
HARW	Harwich	15 November 2015	Pebble beach	42	0	51.9348	1.2813	0
HELG	Helgoland	15 January 2016	Harbour	48	0	54.1760	7.8945	47
HORN	Horns Rev	10 June 2015	Wind farm	67	0	55.4789	7.8110	19
K10B	K10‐B	1 October 2014	O&G platform	48	27	53.3626	3.2539	104
K9A	K9‐A	27 August 2014	O&G platform	48	0	53.5202	3.9925	66
L10G	L10‐G	08 June 2014	O&G platform	48	10	53.4904	4.1952	53
L15A	L15‐A	5 June 2014	O&G platform	48	6	53.3295	4.8302	11
LIMF	Limfjorden	16 June 2014	Longlines	48	2	56.7830	8.9110	0
LISB	Lisbon	14 February 2015	Harbour	37	0	38.7635	−9.0926	0
PAAL	Texel	29 June 2014	Breakwater	48	0	53.0118	4.7083	0
Q13A	Q13‐A	28 May 2014	O&G platform	48	0 to 7	52.1911	4.1361	13
SCHV	Scheveningen	8 July 2014	Breakwater	48	0	52.0987	4.2582	0
SYLT	Sylt	3 June 2014	Breakwater	48	0	55.0216	8.4403	0
WZA	Wadden Sea A	30 April 2014	Intertidal mussel bed	24	0	53.4521	6.3042	0
WZB	Wadden Sea B	6 May 2014	Subtidal mussel bed	24	2	53.4600	6.3583	0

Note

From left to right: abbreviated name of sample, full name of sample, sampling date, structure type, sample size (number of specimens genotyped), sampling depth, position in decimal degrees WGS1984 (latitude and longitude) and distance to the nearest coastline.

Between 50 and ~100 individuals were sampled randomly at every location and stored at −20°C or in 70% ethanol to be transported to the laboratory. All samples were then cleaned of marine growth and placed at −80°C for long‐term storage.

#### 2.2.1 Molecular methods

From each sample, between 24 and 67 specimens were selected randomly and genomic DNA was isolated from the adductor muscles by using a 96‐well genomic DNA extraction kit according to the manufacturer's protocol (FAVORGEN Biotech Corp). DNA concentration was measured on a Nanodrop and diluted to 10 ng/μl. Two multiplex sets of four markers were used (set1: Med367, Med379, Med722 and Med733; set2: Med737, Med740, Med747 and Me15/16). Markers were used individually in the PCR (polymerase chain reaction), pooled per set and analysed on an ABI3730 DNA Analyser. Seven markers were microsatellite loci as described by Lallias, Stockdale, and Boudry (2009) and the Me15/16 locus targeted a part of the adhesive protein gene, which partially discriminates between *M. edulis*, *M. galloprovincialis* and *M. trossulus* (Inoue, Waite, Matsuoka, Odo, & Harayama, 1995). Marker information, PCR conditions and multiplex conditions are detailed in Table 2. The GeneScan 500 LIZ marker was used as an internal marker. Allele calling was performed in genemapper version 3.7 (Applied Biosystems 2004).

**Table 2:** Marker characteristics

Marker	Dye	Temperature (°C)	Size range (bp)	Dilution	Reference
Set 1					
Med 367	VIC	58	190–290	250×	Lallias et al. (2009)
Med379	NED	60	140–230	125×	Lallias et al. (2009)
Med722	PET	55	150–270	125×	Lallias et al. (2009)
Med733	FAM	58	140–270	83.3×	Lallias et al. (2009)
Set 2					
Med 737	VIC	58	130–280	250×	Lallias et al. (2009)
Med 740	FAM	55	150–280	125×	Lallias et al. (2009)
Med 747	NED	55	100–320	125×	Lallias et al. (2009)
Me15/16	PET	55	120–200	83.3×	Inoue et al. (1995)

Note

From left to right: summary of all markers used in this study, fluorescent dye, annealing temperature, allele size range, PCR product dilution in multiplex set and reference of marker used.

### 2.3 Connectivity calculated by particle tracking models

The transport of mussel larvae between sampled subpopulations in the North Sea was modelled using two Delft3D software modules: FLOW and PART (Deltares, 2016b, 2016a). The PART module was able to simulate midfield water quality and particle tracking, based on a hydrodynamic forcing output from the other Delft3D module, FLOW.

#### 2.3.1 Hydrodynamical model

The hydrodynamical model applied was Delft3D‐FLOW, which solves the unsteady shallow‐water equations in three dimensions. The model incorporates a large number of processes, such as wind shear, wave forces, tidal forces, density‐driven flows, stratification, atmospheric pressure changes, air temperature, and the exposure and inundation of intertidal flats. The large number of processes included in this module means that Delft3D‐FLOW can be applied to a wide range of environments (e.g., river, estuarine, coastal and marine areas; Lesser, Roelvink, Kester, & Stelling, 2004). Flow equations were solved on a curvilinear grid consisting of 8,710 computational elements (Roelvink, Jeuken, van Holland, Aarninkhof, & Stam, 2001). The vertical resolution of the model was 10 water layers using a sigma‐coordinated approach (i.e., proportional to water depth; Stelling & van Kester, 1994). Hydrodynamic transport was computed using detailed bathymetry and open boundary forcing based on tidal constituents. The model was forced using meteorological data from the High Resolution Limited Area Model (KNMI, 2015), which comprised two horizontal wind velocity components (at 10 m above mean sea level) and other atmospheric variables such as air pressure and temperature, archived every 6 hr. The freshwater discharges from 18 rivers were included in the model; seven of these discharges varied temporally (daily averages) and 11 were constant (based on long‐term averages). This model is described in detail in Erftemeijer, Beek, Bolle, Dickey‐Collas, and Los (2009).

**Figure 2:**
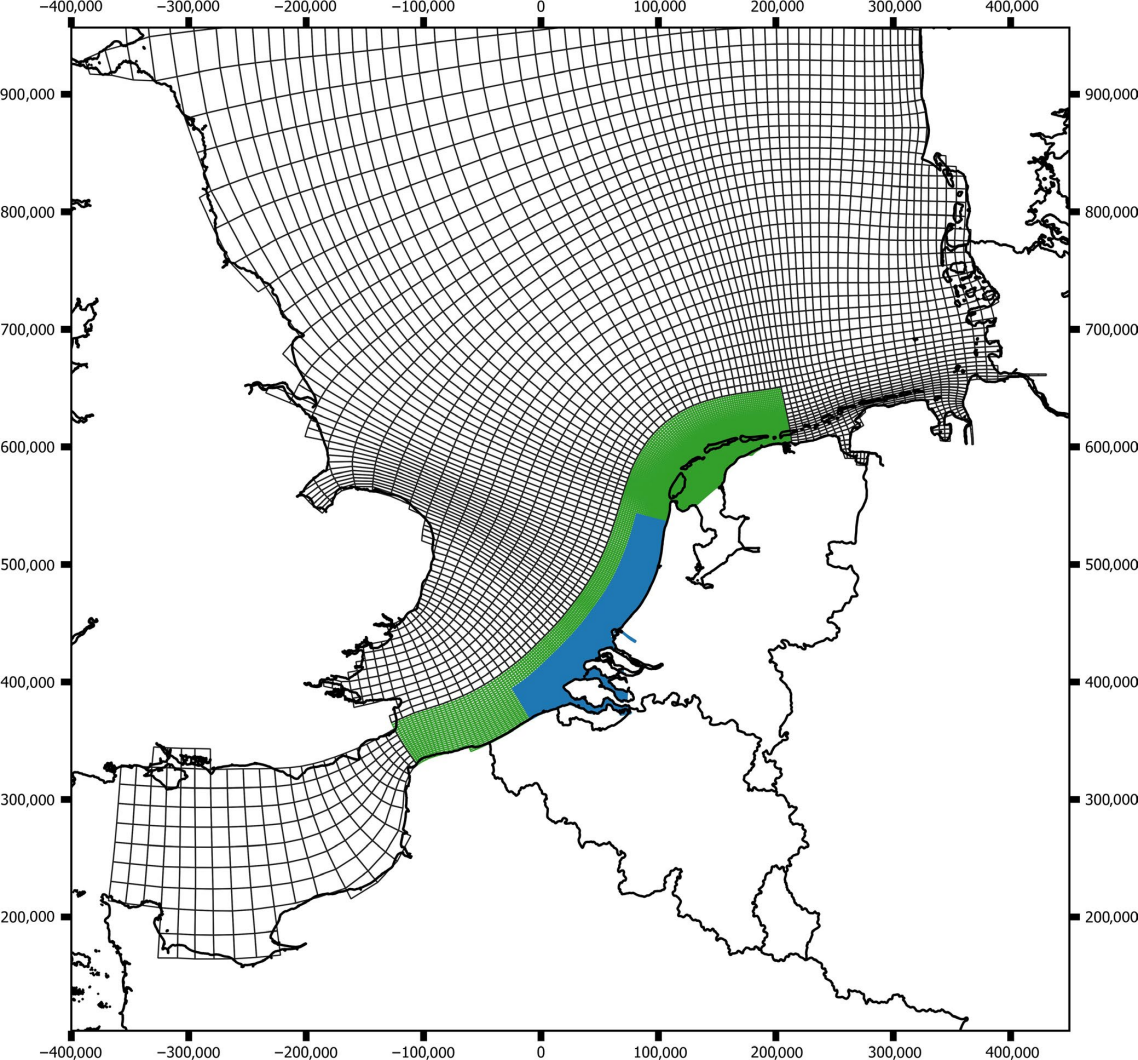
Hydrodynamic model grid. Overview of the ZUNOGROF grid covering the southern North Sea (white cells), and the high‐resolution ZUNO DD grid in the Dutch coastal and inshore areas. Coloured areas: subdomains of the ZUNO DD model, with a higher grid resolution in the Dutch coastal zone and the Wadden Sea. Further explanation is given in the text [Colour figure can be viewed at http://www.wileyonlinelibrary.com/]

Two grid layouts covering the southern North Sea (including the Wadden Sea) were used in this study: a moderately fine grid (ZUNOGROF) and a domain decomposition model grid (ZUNO‐DD) with a much higher grid resolution in the Dutch coastal zone and the Wadden Sea. The subdomains of the ZUNO DD model are displayed in Figure 2 using different colours and are used to increase spatial resolution for hydrodynamic results in these areas. In coastal and inshore (estuarine, lagoonal) systems, the spatial variability in hydrodynamic forcing is much higher. To enable the simulation of these processes, the areas near the Dutch coast and in the Wadden Sea have been given a much higher horizontal grid density, so with smaller grid cells per surface unit. This provides for a much better simulation of the hydrodynamic processes in such areas. These spatial differences in resolution in the Dutch coastal waters and the Wadden Sea have been given a different colouring in Figure 2.

#### 2.3.2 Particle transport model

Particle tracking models are often used in environmental modelling (Broekhuizen, Lundquist, Hadfield, & Brown, 2011; North et al., 2008; Postma, Beek, Boogaard, & Stelling, 2013). Here, the Delft3D‐PART module was used to calculate larval transport across the southern North Sea. Delft3D‐PART is a random walk PTM, based on the principle that movement of dissolved substances in water can be described by a limited but potentially large number of discrete particles that are subject to advection due to currents and by horizontal and vertical dispersion. The movement of the particles in the model consists of two steps: advection, which is driven by the FLOW results; and dispersion, which is a stochastic random walk process. In addition, the horizontal and vertical movement of the particles can be adjusted to account for swimming preferences or changes in buoyancy. Particle tracking allows water quality processes to be described in a detailed spatial pattern, resolving subgrid concentration distributions. Delft3D‐PART is shown to be locally mass conservative (Postma et al., 2013).

#### 2.3.3 Modelling set up

Delft3D‐FLOW calculated the hydrodynamic conditions for the southern North Sea for two consecutive years, 2004 and 2005. Meteorological conditions are important to determine the geographical destination of particles such as larvae, superimposed on the regular transport conditions in the southern North Sea. At the time of analysis, there were no hydrodynamic modelling results available for the years of sampling, 2014–2016. Instead, two model runs from 2004 and 2005 were used. Although there is large variation in climatic forcing between years, we chose these two years because of the overall comparable (southern) North Sea wind patterns during the months we used for our PTM (i.e., March–June). The 2 years were especially comparable with regard to wind directions and wind force. We could only roughly assess this comparability from the meteorological data available to us (Royal Dutch Meteorological Institute); we did not perform a detailed analysis. We assume that through this approach the differences between the hydrodynamic circulation patterns in the (southern) North Sea between these two periods is less than when random years would have been used. For the Delft3D‐PART model, each sampling location acted as an origin point of larvae, and the density of the larvae throughout the consecutive months at the other locations (destinations) was calculated. Larval release was started on day 59 (1 March) of each year, from the top layer in the model. Both the timing and the period of particle release were based on the general reproductive timing and pelagic larval stage period of *M. edulis*. Mussels start spawning in early spring, based on temperature of the water. Variation of spawning timing may result from the variation in temperature increase between years; in the southern North Sea this usually starts in March, and peaks in April/May or even later (De Vooys, 1999). Time from spawning of mussel larvae to settlement is around 3 months (Widdows, 1991). It was further assumed that the larvae are largely passive and that they are transported only by advection and general dispersion. They were also considered neutrally buoyant. Each origin released 1,489 particles per 30 min, for 14 days (1,000,001 particles in total), after which no further release of particles occurred. The program calculated the horizontal transport and vertical dispersion to lower layers with time steps of 30 min for 70 days. Although mortality of *M. edulis* larvae is expected to be very high (Barker Jørgensen, 1981), this study did not include this aspect in the Delft3D‐PART model. The purpose of the study was to assess the maximum success of larvae settling at a specific destination, which was assumed to be proportional to the total number of larvae arriving at a specific destination throughout the 70 days. The total number of arriving particles from each source–destination combination was calculated and combined in a particle matrix.

### 2.4 Data analysis

#### 2.4.1 Hybrid removal

A mix of alleles of *M. galloprovincialis* and *M. trossulus* was observed in our data at the partially species diagnostic locus Me15/16, and therefore we estimated the hybrid index *h* for each individual using a maximum likelihood approach that estimates the proportion of alleles inherited from one of two hybridizing species (Buerkle, 2005; Buerkle & Lexer, 2008). This approach was applied as implemented in the r package introgress (Gompert & Alex Buerkle, 2010). The samples from Lisbon (*M. galloprovincialis*) and Limfjorden (*M. edulis)* were used as parental reference samples and locus Med379 was not taken into account due to high levels of missing data for several samples. All individuals identified as nonpure *M. edulis* (*h* < 1.0) were omitted from subsequent analyses, as this aspect would have affected the non‐neutral influences on connectivity inferences.

#### 2.4.2 Population genetic statistics

Population‐level analyses included pairwise Wright's *F*_ST_ calculations (Wright, 1943, 1965) and analysis of molecular variance (AMOVA), performed using genalex 6.5 (Peakall & Smouse, 2006, 2012), r (R Core Team, 2015) and rstudio (RStudio Team, 2015). Locus Me15/16 was not included in the analysis. Pairwise *F*_ST_ was assumed to be significantly different only when *p* ≤ .0001 (which is *p* ≤ .05 with Bonferroni correction for 351 pairs). microchecker version 2.2.0.3 (van Oosterhout, Hutchinson, Mills, & Shipley, 2004) was used to test for the presence and estimate the frequency of null alleles. Allelic richness was estimated using the popgenreport package in r (Adamack & Gruber, 2014) and other descriptive statistics (number of alleles [*N*_a_], effective number of alleles [*N*_e_], number of private alleles [*N*_p_], observed heterozygosity [*H*_O_], expected heterozygosity [*H*_E_] and mean allelic richness [Ar]) using genalex 6.5. Isolation by distance was tested for by comparing the linear distance matrix among sampling locations with the pairwise *F*_ST_‐matrix and performing a Mantel test with 10,000 permutations. This was done using the r package fossil version 0.3.7 (Vavrek, 2011). In addition to these population‐level analyses, the possible presence of genetic structure was also tested for by using Bayesian clustering algorithms implemented in the software structure version 2.3.4 (Pritchard, Stephens, & Donnelly, 2000). These algorithms are able to detect structure in the form of subgroups in the sampled individuals without a priori assuming that this structure is organized according to sampling location. With structure, no‐admixture models were run with burn‐in times of 100,000 and 100,000 repeats after burn‐in with number of groups (*K*) ranging from 1 to 25.

#### 2.4.3 Isolation with migration analysis

Effective gene flow between sampling locations was estimated for all possible pairs, using the coalescent approach implemented in the software 'Isolation with Migration' (ima2p; Hey & Nielsen, 2004; Sethuraman & Hey, 2016). The ima2p model consists of subpopulations that are simulated backwards in time towards merging sometime in the past. Parameters for time since population divergence *t*, population sizes *q* and bidirectional migration rates *m* are estimated by a Metropolis‐coupled Markov chain Monte Carlo process. To avoid overfitting the data we only ran simulations for pairs of samples. Coalescent simulation runs consisted of 10 Markov chain Monte Carlo chains with geometric heating (setting parameters ha = 0.99, hb = 0.75) and 10 million steps after an initial burn‐in period of 5 million steps. The parameters estimated and parameter ranges examined (in coalescent units) were three population sizes *q* (ancestral and two populations ensuing from population subdivision, range examined 0–100), two migration rates after population subdivision (both directions, range examined 0–1) and time since population subdivision *t* (range examined 0–10). Convergence of estimated parameter distributions was ensured by: checking effective sample size (ESS) values, autocorrelation values and chain swapping, examining trend line plots for absence of trends, and comparing parameter estimates generated from the genealogies produced during the first and second half of runs. A mutation rate for the seven microsatellite loci of 0.001564 per generation was assumed, following the methodology of Luttikhuizen et al. (2018). Migration rates among sampled locations were visualized by chord diagrams using the r package circlize version 0.4.3 (Gu, Gu, Eils, Schlesner, & Brors, 2014).

The fit of the pairwise isolation‐with‐migration models to the data and the underlying processes was assessed by comparing the distribution of estimated migration rates with that of the pairwise *F*_ST_ values directly estimated from the microsatellite data. Pairwise *F*_ST_ values were converted to 2*N*_e_ *m* values following the infinite island model approximation by Wright (1943) in which *F*_ST_ = (1 + 4*N*_e_ *m*)^–1^. Correspondence between 2*N*_e_ *m* values stemming from *F*_ST_ estimation and from isolation‐with‐migration simulations was compared using Pearson's rank correlation. Correlation was performed with the average between the two (bidirectional) migration rates stemming from isolation‐with‐migration simulations.

#### 2.4.4 Migration and population differentiation versus arriving particles

To validate our hypothesis, we tested if the hydrography could partly determine connectivity of mussel populations growing on offshore structures in the southern North Sea. Thus, we tested the prediction that the number of arriving particles in the hydrogeographical model correlates with the genetic population differentiation statistic *F*_ST_ as well as with migration rate as inferred from coalescent simulations. The statistical significance of these correlations was assessed by a Mantel test (Mantel, 1967). Most implementations of the Mantel test assume that matrices whose correlation is to be tested are symmetrical; that is, when in a matrix *M* for all cells *m_i,j_* = *m_j,i_* where *i* equals row number and *j* equals column number. However, this is not generally true as tidal currents that are expected to drive connectivity differ in current speed between ebb and flow (McCave, 1971), and therefore in this work, the particle and migration rate matrices were asymmetrical. To determine the significance of the correlation between the isolation matrix and the particle matrix, we implemented the Mantel test ourselves in r. The Mantel test calculates the fraction of random permutations of the matrices that have a higher correlation compared with the original matrices. If the two matrices are unrelated, we expect half of the permutations to have a higher correlation (and the other half to a have a lower one). Here, correlation is the Pearson product‐moment correlation coefficient. The number of permutations used was 10,000.

## 3 RESULTS

The number of alleles per locus varied between three and 69 (Table 3) with between 8% and 28% null alleles. The resulting data set had 23.14% null alleles. These high numbers of null alleles are in agreement with previously published data on microsatellite analyses in marine bivalves and gastropods (Bierne et al., 2003; Carlsson, 2008; Lemer, Rochel, & Planes, 2011; Panova, Mäkinen, Fokin, André, & Johannesson, 2008).

**Table 3:** Information per locus

Locus	*N*_alleles_	Null alleles	*H*_O_	*H*_E_
Med367	52	0.0788	0.756	0.879
Med379	45	0.1947	0.368	0.568
Med722	53	0.2370	0.609	0.894
Med733	37	0.2099	0.480	0.786
Me15/16	3	n.a.	n.a.	n.a.
Med737	61	0.1307	0.819	0.920
Med740	61	0.1054	0.544	0.808
Med747	69	0.2729	0.588	0.897

Note

Summary table containing (from left to right) the locus name, number of alleles observed in the full data set, fraction of null alleles (average between reference samples from Limfjorden [Denmark] and Lisbon [Portugal]), observed heterozygosity *H*_O_; expected heterozygosity *H*_E_ (averaged over samples for data excluding hybrids). Locus Me15/16 was only used for initial screening for the presence of hybrids and not for subsequent intraspecific analyses.

Abbreviation: n.a., not applicable.

**Figure 3:**
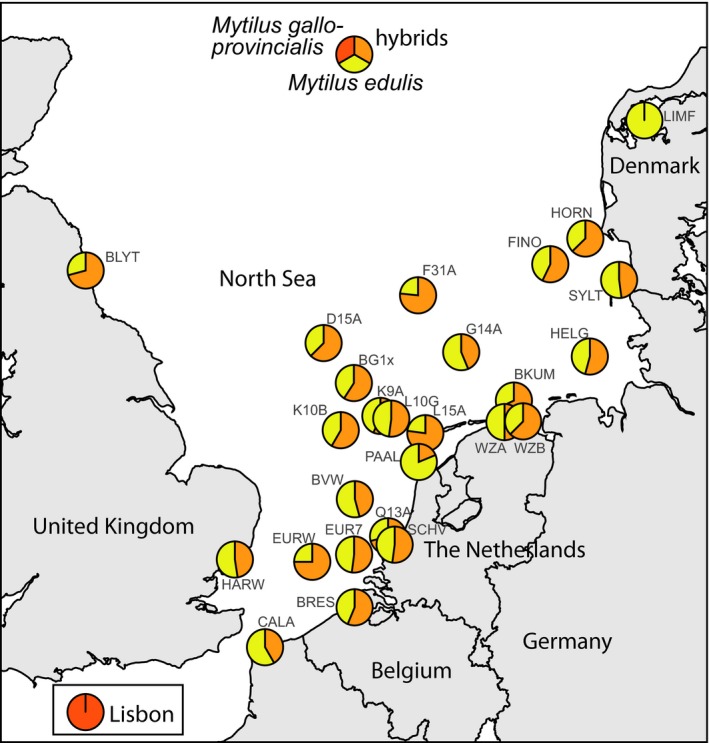
Distribution of *Mytilus galloprovincialis* hybridization. Pie charts indicating the relative amounts of pure *Mytilus edulis* and hybrids with introgression from *M. galloprovincialis* at sampling locations, based on hybrid index *h* estimated from seven microsatellite loci. Individuals with *h *< 1.0 were classified as hybrids. Reference samples were Lisbon, Portugal (bottom left insert) and Limfjorden, Denmark [Colour figure can be viewed at http://www.wileyonlinelibrary.com/]

**Figure 4:**
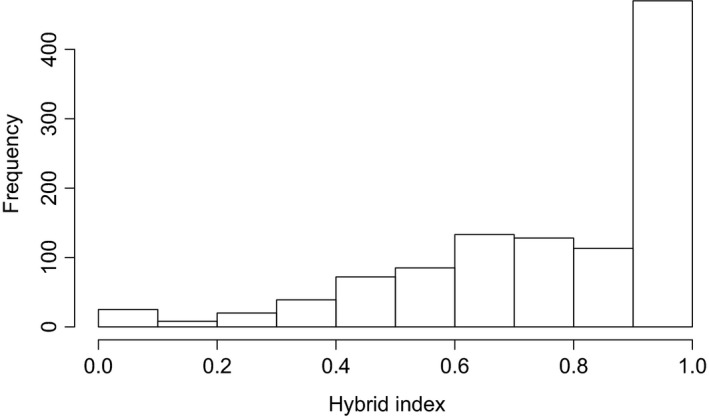
Frequency distribution of hybrid index. Histogram of hybrid index *h* variation found among samples of *Mytilus* spp. in the southern North Sea region (*N* = 1,093 individuals). Individuals with *h* = 1 may be considered as pure *Mytilus edulis* as compared to reference sample from Limfjorden, Denmark; and those with *h* = 0 as pure *Mytilus galloprovincialis* compared to Lisbon, Portugal. A range of hybrid genomic backgrounds was detected, with a majority pure *M. edulis* (53%) and, among hybrids, a bias towards stronger introgression from *M. edulis*

Results from the species diagnostic locus Me15/16 indicated the presence of nonpure *Mytilus edulis* genetic material between our samples; a single *M. trossulus*‐type allele (allele 170 bp) was observed as a heterozygote in a Scheveningen sample (sample SCHV) and a large number of the *M. galloprovincialis*‐type allele were observed as homozygotes and heterozygotes (allele 126 bp). The introgression in *Mytilus* spp. is pervasive and therefore this single locus was not sufficient for detecting a hybrid status of individuals, but a hybrid index proved to be more informative (Figure 3). Using Lisbon (*N* = 37) and Limfjorden samples (*N* = 48) as reference samples in the hybrid analysis, a wide range of hybrid indices was detected among the remaining individuals (Figures 3 and 4). Omitting all nonpure *M. edulis* individuals resulted in a remaining total of 579 genotyped pure *M. edulis* specimens for further analysis. Even in the pure *M. edulis* data set variation was high, with numbers of alleles per sample ranging from 8.7 to 21, effective numbers of alleles per sample from 6.2 to 12.5 and allelic richness from 8.1 to 13.2 (Table 4). The large numbers of alleles also corresponded with a wide range in numbers of private alleles, from 0 in four different samples to 11 in sample K10B (Table 4).

**Table 4:** Descriptive genetic statistics per sample

Sample	*N*_a_	*N*_e_	*N*_p_	*H*_O_	*H*_E_	*A*_r_
BG1x	8.86 (2.13)	6.22 (1.47)	3	0.53 (0.11)	0.69 (0.13)	8.1
BKUM	10.71 (0.99)	8.45 (0.72)	2	0.66 (0.08)	0.88 (0.01)	9.8
BLYT	14.71 (1.3)	10.01 (1.03)	4	0.62 (0.05)	0.89 (0.01)	11.2
BRES	17.43 (3.43)	11.15 (2.31)	4	0.62 (0.11)	0.78 (0.13)	13.2
BVW	17.57 (3.85)	10.54 (2.66)	6	0.57 (0.11)	0.77 (0.13)	12.3
CALA	13.29 (1.06)	9.67 (1)	3	0.71 (0.04)	0.89 (0.02)	11.0
D15A	17 (0.82)	11.76 (0.76)	2	0.62 (0.06)	0.91 (0.01)	12.2
EUR7	14.57 (1.89)	9.16 (1.33)	4	0.68 (0.09)	0.87 (0.02)	10.6
EURW	11.29 (1.02)	6.83 (0.71)	0	0.73 (0.09)	0.84 (0.02)	9.6
F31A	8.86 (1.83)	6.33 (1.55)	1	0.57 (0.12)	0.67 (0.14)	9.5
FINO	11.57 (1.81)	9.65 (1.51)	3	0.65 (0.07)	0.87 (0.04)	11.4
G14A	18.29 (3.72)	10.88 (2.9)	8	0.51 (0.1)	0.78 (0.13)	12.8
HARW	21 (2.24)	12.48 (2.11)	6	0.65 (0.07)	0.9 (0.02)	12.9
HELG	18.29 (1.38)	11.66 (1.46)	2	0.67 (0.07)	0.9 (0.01)	12.5
HORN	15.14 (2.5)	8.13 (1.15)	0	0.64 (0.05)	0.86 (0.03)	10.7
K10B	20 (1.7)	12.53 (1.55)	11	0.75 (0.04)	0.91 (0.01)	12.7
K9A	12.86 (2.52)	7.65 (1.63)	4	0.52 (0.09)	0.75 (0.13)	10.7
L10G	13.86 (2.61)	9.03 (1.99)	2	0.5 (0.1)	0.77 (0.13)	11.5
L15A	9.71 (2.36)	5.77 (1.64)	2	0.4 (0.11)	0.7 (0.12)	9.0
PAAL	17.71 (2.63)	10.34 (1.12)	3	0.55 (0.09)	0.9 (0.01)	11.1
Q13A	13.14 (3.12)	8.66 (2.3)	0	0.4 (0.11)	0.75 (0.13)	11.3
SCH	16.29 (2.03)	10.73 (1.62)	1	0.67 (0.08)	0.89 (0.02)	11.6
SYLT	11.71 (1.66)	8.08 (1.03)	2	0.51 (0.08)	0.86 (0.02)	10.4
WZA	12.29 (2.25)	8.82 (1.72)	0	0.57 (0.11)	0.77 (0.13)	11.6
WZB	10.71 (2.3)	7.72 (1.71)	0	0.55 (0.11)	0.75 (0.13)	10.5

Note

Number of alleles (*N*_a_), effective number of alleles (*N*_e_), number of private alleles (*N*_p_), observed heterozygosity (*H*_O_), expected heterozygosity (*H*_E_) and mean allelic richness (*A*_r_).

AMOVA revealed significant population structure among the 25 samples (*F*_ST_ = 0.096, *p* = .001, Table 5). The AMOVA estimated *F*_IS_ at 0.410 (*p* = .001) and *F*_IT_ at 0.466 (*p* = .001). Pairwise *F*_ST_ values (Table S1) ranged between 0 and 0.17 with an average of 0.06. In total, 273 out of 351 pairs were significantly different from zero. Isolation by distance of linear distance against pairwise *F*_ST_ was not significant (Mantel test, *r *= .078, *p* = .22; Figure S4). The analysis of possible population patterns without a priori classification (program structure) showed that there was no clear support for a particular number of subgroups within the data. This was indicated by the gradual increase of *X*|*K* (the likelihood of the data given number of groups *K*) with number of groups *K* (Figure S5), which is here cautiously interpreted as either no support for obvious subgroups (Kalinowski, 2011; Porras‐Hurtado et al., 2013), or, alternatively, that many small groups exist with subtle differences between them. Figure S6 shows structure bar plots for *K* = 2–7.

**Table 5:** Results of analysis of molecular variance

	*df*	SS	MS	Variance component	Percentage
Among samples	24	439	18.28	0.307	10
Among individuals	554	2,274	4.11	1.193	37
Within individuals	579	996	1.71	1.719	53
Total	1,157	3,708		3.219	100

Note

Partitioning of genetic variation within and between individuals from 25 samples of mussels *M. edulis* in the southern North Sea.

Abbreviation: *df*, degrees of freedom; MS, mean squares; SS, sum of squares.

**Figure 5:**
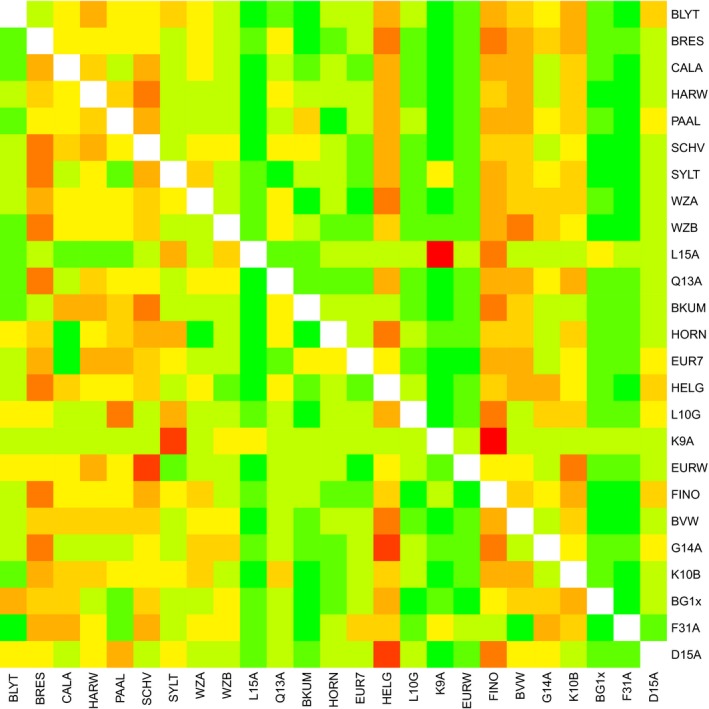
Heat map showing migration rates among sampled locations. Estimated migration rate among all locations, red = high migration, green = low migration. *x*‐axis: origin population, *y*‐axis: destination population. Locations have been ordered in increasing distance to the nearest shore (Table 1) [Colour figure can be viewed at http://www.wileyonlinelibrary.com/]

Inferred migration per generation between sampling locations (Table S2; Figure 5) ranged between 0.007 and 37.16 individuals per year with an average of 10.78. Average migration rates to subpopulations BVW, Calais, Helgoland and Scheveningen were the highest with values >14. With values of <4, K9‐A and L15‐A had the lowest average migration rates from other sampled stations. The highest migration rates (>30) were from Breskens to Helgoland (37), Scheveningen (33) and Wadden Sea location B (33), from Helgoland to Breskens (32), BVW (35), Calais (30) and G14‐A (34), from BVW to Helgoland and from Scheveningen to Harwich (30).

Migration rates estimated using pairwise isolation‐with‐migration simulations correlated significantly with migration rates estimated directly from pairwise *F*_ST_ values calculated from the microsatellite data (Pearson's *r* = .189, *p* = .0012). Overall, migration rates estimated from pairwise *F*_ST_ values were lower than those estimated by isolation‐with‐migration simulations (median 5.52 and 9.26, respectively) and the distribution of the former was also more skewed (Figure S7).

**Figure 6:**
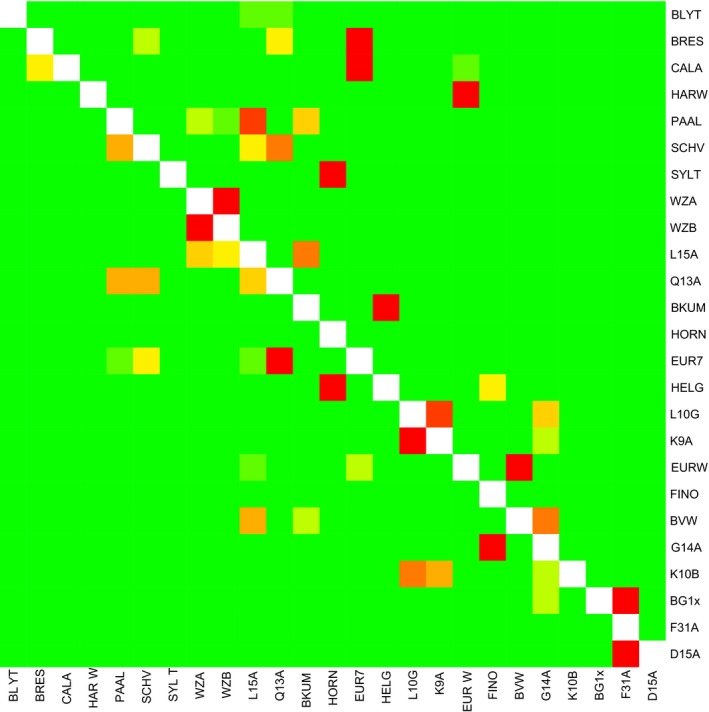
Heat map showing particle exchange rates among sampled locations. Estimated particle exchange rate among all locations, red = high particle arrival, green = low particle arrival. *x*‐axis: origin population, *y*‐axis: destination population. Locations have been ordered in increasing distance to the nearest shore (Table 1) [Colour figure can be viewed at http://www.wileyonlinelibrary.com/]

The number of arriving particles in the hydrodynamic model showed high variation and many nonconnected locations (Table S3; Figure 6). From a total of 625 combinations (including local retention), 478 combinations showed no connection at all. Highest local retention was found in coastal locations BRES, CALA, HARW, WZA and WZB, whereas offshore southern locations BG1x, BVW, EUR7 and EURW as well as offshore K10‐B and nearshore Q13‐A showed low local retention. Some of the sites, such as WZA and WZB, were very highly connected, and geographically these areas are in close proximity. Particle movement from EURW to BVW and well as from EUR7 to Q13‐A sites was intermediate, whereas most of the other locations showed low numbers of arriving particles. BLYTH was the only location that had no connection with any of the other locations.

The Pearson product‐moment correlation between pairwise *F*_ST_ and arriving particles was −0.16 (*p* = .06), indicating that *F*_ST_ was low between pairs with high particle exchange rates. The correlation between migration rate and arriving particles was .002 (*p* = .13). The effective travel distance and direction of particles thus explains part of the variation in pairwise *F*_ST_ but not for migration rate.

**Figure 7:**
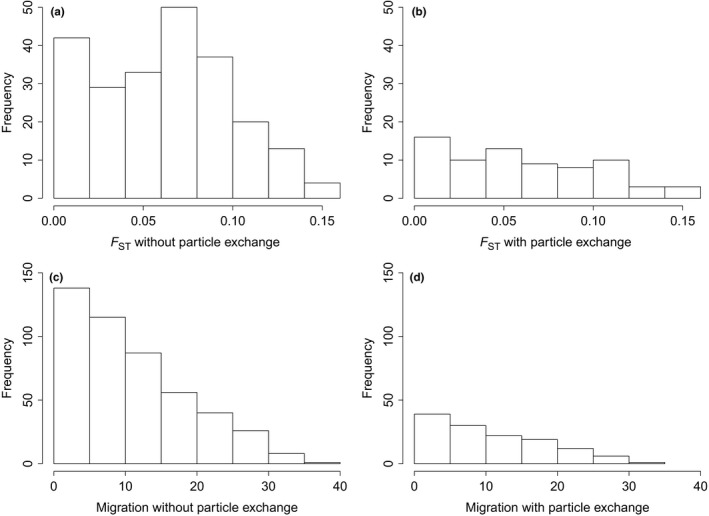
Histograms of pairwise *F*_ST_ frequencies and migration per generation. (a) Pairwise *F*_ST_ frequencies of sample combinations that exchange no particles in the particle tracking models (PTMs). (b) Pairwise *F*_ST_ frequencies of sample combinations that do exchange particles in the PTM. (c) Migration per generation between sample combinations that exchange no particles in the PTM. (d) Migration per generation between sample combinations that do exchange particles in the PTM

Sample combinations without particle exchange had a 35% higher pairwise *F*_ST_ compared to combinations with particle exchange (Figure 7a,b; *p* = .10, Kolmogorov–Smirnov test), being in line with the Mantel test. For the migration per generation between subpopulation combinations with and without particle exchange this difference was not apparent (Figure 7c,d; *p* = .44).

## 4 DISCUSSION

This study contributes with novel results to the understanding of the marine stepping‐stone theory (Adams et al., 2014; Henry et al., 2018). Although PTM results predicted that *Mytilus* larvae should be unable to reach locations >85 km from coastal mussel populations, *Mytilus* spp. were found at all inspected locations, up to 181 km from the nearest coastline. Colonization of such far from shore locations by the mussels would require an adult population within a radius of 85 km. However, our analysis was unable to show that migration rates followed a pattern explained by modelled particle connectivity. Furthermore, although we found an indication that *F*_ST_ was lower between connected locations, isolation by distance analysis showed no increase in isolation with increasing distance. Finally, we did not find clear structure among the populations. For example, many of the investigated locations were isolated from nearby locations.

Although mussels were present far offshore, we did not show them to originate from the intermediate stepping‐stone locations investigated by us. The mussels colonized the available structures from unknown sources that placed larvae at offshore locations in an unknown manner. Then, after successful colonization, most populations had no—or only limited—exchange with the nearby locations of the study, as suggested by the PTM. Following generations may have migrated to more proximate locations but exchange with locations further away was not apparent so no isolation by distance could be shown at a larger scale.

Although *M. edulis* can be observed in deeper waters (Table 1), it is most abundant in shallow waters as its presence is limited by predation in deeper waters (Langhamer, 2010). Consequently, large reproductive individuals of *M. edulis* are effectively absent on natural offshore reefs without intertidal zones (Coolen, Bos, et al., 2015). The addition of many intertidal surfaces to the North Sea by artificial structures provides novel habitats for *M. edulis* (Krone et al., 2013). Without these shallow hard substrates, *M. edulis* would be unlikely to survive in offshore North Sea waters.

At all locations in the North Sea, hybridization between *M. edulis* and *M. galloprovincialis* was observed. This extends the known hybrid zone between these taxa further north than hitherto described (Bierne et al., 2003; Fraïsse et al., 2014). Possibly, as earlier studies were restricted to nearshore locations, artificial offshore hard substrates seem to house more mussel hybrids than nearshore substrates. Alternatively, *M. galloprovincialis* may have spread northwards in recent years. The latter hypothesis awaits new nearshore genotyping of blue mussels, but the fact that our own nearshore sampling sites were found to contain many hybrids (Figure 3) is indicative of a possible northward spread of *M. galloprovincialis*. The single individual carrying a single *M. trossulus* allele originated from a harbour (Scheveningen) and may therefore be a descendant of mussels imported by ships as vector.

After removal of hybrids, 53% of the specimens in the data remained that were considered pure *M. edulis*. Based on these pure *M. edulis* specimens, the number of particles modelled to be exchanged between structures correlated with the pairwise *F*_ST_ values of *M. edulis* (correlation −.16, *p* = .06). Even though our analyses probably included specimens of several different year classes, which may be expected to result from recruitment events under different hydrodynamic regimes, we nevertheless detected a correlation between the pairwise *F*_ST_ and arriving particle matrices.

Considering the young age of the artificial substrates we studied, and hence also of the populations of blue mussels living on them, the level of population genetic differentiation between many of the samples was remarkably high. On an evolutionary timescale these populations have been established extremely recently, as all offshore locations investigated were less than 50 years old. The range in population sizes must have been high, varying between an estimated <50,000 on a fully overgrown buoy, 50,000,000 on a medium‐sized platform and billions on coastal locations, following generalized installation sizes and average *M. edulis* densities on offshore structures (Coolen et al., 2018; van der Stap, Coolen, & Lindeboom, 2016). Population genetic differentiation requires a number of generations on the order of the effective population size (the number of successfully reproducing individuals in a random mating population) to develop into an equilibrium state. The small initial population sizes may have accelerated the process of differentiation. We carried out coalescent analyses of isolation‐with‐migration that do not assume equilibrium between drift and migration (Hey & Nielsen, 2007; Sethuraman & Hey, 2016). The migration analysis, however, was found to not corroborate the correlation between genetic differentiation and hydrogeographical modelling. Founder effects (Nei, Maruyama, & Chakraborty, 1975) may have further obscured patterns in migration rates. If rare events have caused colonization of the structures from nearby but unconnected populations, the founder effect would cause the newly established population to seem isolated even though it originated from neighbouring populations. This founder effect is likely to increase after marine growth removal, as performed every 2–5 years on platforms and buoys (Coolen et al., 2018), which provides clean surfaces for new colonizers, resetting the age of the subpopulation every few years (Kerckhof & Cattrijsse, 2001).

It is possible that the pairwise population comparisons as modelled in ima2p (Hey & Nielsen, 2004; Sethuraman & Hey, 2016) did not fit well enough to the actual processes driving the situation in the field, causing uncorrelated results in our migration rates analysis. However, we did find a correlation between migration rates from coalescent analysis and rates based on *F*_ST_. This indicates that modelled migration rates fit the actual processes underlying genetic differentiation to some extent. Part of the colonization of offshore artificial hard substrates may have been the result of exceptional events (e.g., abnormal weather patterns, followed by isolation). Consequently, settlement on an offshore object by drifting mussel larvae might be rare. If such colonization events are rare and mussel stocks on offshore installations are isolated from each other most of the time, estimated migration rates would not correlate with total arriving particles as predicted from hydrogeographical models. This would be reflected in the observed patterns of genetic differentiation, underlining the rare colonization processes.

Blue mussels also occur on the hulls of ships and can be attached to flotsam (Apte, Holland, Godwin, & Gardner, 2000; Hopkins & Forrest, 2010; Thiel & Gutow, 2005). The introduction of larvae to offshore structures may therefore have been facilitated by anthropogenic activities. A potential source of larvae may have been jack‐up rigs that are used near offshore installations such as platforms. These jack‐ups can be covered in marine growth with high abundance of mussels (J.W.P. Coolen, personal observation). They are placed offshore near installations for various periods of time and are then relocated to other work sites offshore. Adult mussels on these jack‐up rigs may spawn and cause colonization by mussels that are not closely related to nearby populations.

Mussels have been transported historically between the Wadden Sea and waters near Breskens (Muehlbauer et al., 2014), providing higher genetic connectivity than would be expected from isolation by distance. The resulting genetic patterns may have obscured connectivity signals of the investigated subpopulations. This effect is supported by the low *F*_ST_ for WZA and BRES, which are 380 km apart. These exchange activities should be addressed in future analyses, for example by following the methodology of Hernawan et al. (2017).

A further reason that may have led to the noncorrelation of estimated migration rates and modelled number of arriving particles could be that the particle arrival rates between sampling locations did not represent the actual hydrodynamic processes, which are underlying the exchange of larvae between these locations. Accordingly, comparison studies revealed large variations between different PTMs (Hufnagl et al., 2016). Particle models are driven by hydrodynamic models that are driven by meteorological forcing, which may influence tidal movements and residual currents in the North Sea. Changing the meteorological forcing influences the outcome of the model. Furthermore, changing the time of spawning, length of spawning and survival period of larvae as modelled in the PTM is also likely to change the outcome of the model, because usually the weather changes over the season. Our choices are based on an “average” year, without predominant and strong easterly or westerly winds, and outflow from rivers, as well as average data on spawning and development of mussel larvae. Significant changes are therefore only expected in case of strong deviations in wind direction and force, and temperature in the spring. Because the collection of the mussels occurred over various years, during which the spring weather was mostly average, there is no reason to assume other input variables than chosen for the model runs.

Furthermore, many sample combinations were not connected according to the PTMs, resulting in many zeros in the arriving particles matrix. This was probably due to an average distance between locations that was larger than the average distance travelled by larvae. Future research should address this by sampling locations in closer proximity than in the current study. This will decrease the number of location combinations with zero particle exchange and is likely to better describe connectivity.

The stepping‐stone effect possibly contributes to changes in distribution of nonindigenous species (Adams et al., 2014). Here, we used an indigenous species as model organism to test the concept of marine stepping‐stones in the North Sea. In that region, nonindigenous species such as the Japanese amphipod *Caprella mutica* were found at the same offshore energy installations (Coolen et al., 2016, 2018). The spread of native and non‐native species depends largely on reproductive parameters and dispersal strategies. In the North Sea, any stepping‐stone effect will be of most importance for epifaunal species with pelagic larval stages of several weeks (e.g., the oyster *Ostrea edulis* with 10–30 days or the limpet *Crepidula fornicata* with 14–21 days; Berghahn & Ruth, 2005; Shanks, 2009). Species with much shorter pelagic stages may be unlikely to reach the next subpopulation whereas species with very long pelagic larval stages may be capable of bridging large distances and may not need stepping‐stones to colonize distant locations (Reisser, Bell, & Gardner, 2014). Other organisms, such as the nonindigenous *Caprella mutica*, also use floating objects such as ship hulls or debris for dispersal (Thiel & Gutow, 2005), and some species are transported by ballast water (Drake & Lodge, 2004), enabling them to colonize distant locations. This may be the most important factor driving the invasion by some non‐native species (Coolen et al., 2016).

The importance of artificial hard substrates as stepping‐stones remains unclear. They may increase the speed of (future) bio‐invasive events, as they provide valuable habitat in isolated locations for nonindigenous species. However, other factors such as hull fouling and ballast water exchange also strongly affect the distribution of nonindigenous species. For newly built installations, effects need to be evaluated in the context of tens of thousands of other artificial objects already present, including shipwrecks that have been there for more than 100 years (Coolen, Lengkeek, et al., 2015).

In conclusion, artificial structures provide the only suitable habitats for species such as *M. edulis* at far offshore locations in the North Sea. Our results suggest that unexplained and rare colonization events may act together with average migration patterns to shape population genetic patterns and population connectivity. Anthropogenic activities may have influenced genetic exchange between locations, for example via hull fouling on offshore vessels such as jack‐up rigs. More detailed analyses of interannual variability in predicted larval transport patterns and inclusion of historical activities would be desirable. Future research should decrease the distance between modelled locations, to decrease the number of population combinations with zero connectivity in the modelled particle data.

## AUTHOR CONTRIBUTIONS

J.W.P.C., A.R.B., P.C.L.: designed research; performed research; analysed data; wrote the paper. R.C., H.P., L.E.B.: designed research; wrote the paper. F.K.: performed research; analysed data; wrote the paper. D.G.: analysed data; wrote the paper. J.B., S.N.R.B.: wrote the paper.

## Data Availability

All microsatellite data, migration per generation data and particle tracking data are publicly available on Dryad via: https://doi.org/10.5061/dryad.612jm6405.
