# Supplementary material for: Marine stepping‐stones: Connectivity of Mytilus edulis populations between offshore energy installations
Source: Mol Ecol. 2020 Feb 11;29(4):686–703. doi: 10.1111/mec.15364 (PMC7065051; doi:10.1111/mec.15364)
Supplement: Supplementary file 1 [file MEC-29-686-s001.pdf]

Supporting information to:

Marine stepping-stones: Connectivity of *Mytilus edulis* populations between offshore energy installations

Coolen, Joop W.P.[1,2], Boon, Arjen R. [3], Crooijmans, Richard. [4], van Pelt, Hilde[1], Kleissen, Frank [3], Gerla, Daan[1], Beermann, Jan.[5,6], Birchenough, Silvana N.R.[7], Becking, Lisa E.[1,8], Luttikhuizen, Pieternella C.[9]

1. Wageningen Marine Research, P.O. Box 57, 1780 AB Den Helder, The Netherlands.
2. Wageningen University, Aquatic Ecology and Water Quality Management Group, Droevendaalsesteeg 3a, 6708 PD Wageningen, The Netherlands.
3. Deltares, Marine and Coastal Systems, P.O. Box 177, 2600 MH Delft, The Netherlands.
4. Wageningen University, Animal Breeding and Genomics Centre, Droevendaalsesteeg 1, 6708 PB Wageningen, The Netherlands.
5. Alfred Wegener Institute Helmholtz Centre for Polar and Marine Research, Department of Functional Ecology, Am Handelshafen 12, 27570 Bremerhaven, Germany.
6. Helmholtz Institute for Functional Marine Biodiversity, Oldenburg, Germany.
7. Centre for Environment, Fisheries & Aquaculture Science, Pakefield Road, Lowestoft, Suffolk NR33 0HT, United Kingdom.
8. Wageningen University, Marine Animal Ecology Group, De Elst 1, 6708 WD Wageningen, The Netherlands.
9. NIOZ Royal Netherlands Institute for Sea Research, Department of Coastal Systems, and Utrecht University, P.O. Box 59, 1790AB Den Burg, The Netherlands

Corresponding author: Joop W.P. Coolen; joop.coolen@wur.nl

Table S1. Pairwise F_{ST}.

Pairwise F_{ST} between pairs of samples. Locations with F_{ST} in bold differ significantly ($p<0.05$) after Bonferroni correction for 351 pairs. For location abbreviations see table 1.

	BG1x	BKUM	BLYT	BRES	BVW	CALA	D15A	EUR7	EURW	F31A	FINO	G14A	HARW	HELG	HORN	K10B	K9A	L10G	L15A	LIMF	LISB	PAAL	Q13A	SCHV	SYLT	WZA
BKUM	**0.1598**																									
BLYT	**0.1395**	0.0000																								
BRES	**0.0771**	**0.0974**	**0.0800**																							
BVW	**0.0719**	**0.1068**	**0.0835**	0.0000																						
CALA	**0.1127**	0.0000	0.0000	**0.0707**	**0.0702**																					
D15A	**0.1496**	0.0080	0.0000	**0.0840**	**0.0902**	0.0052																				
EUR7	**0.0753**	0.0367	**0.0309**	**0.0304**	**0.0382**	0.0291	**0.0350**																			
EURW	**0.0875**	0.0459	**0.0565**	**0.1033**	**0.1089**	**0.0417**	**0.0632**	**0.0444**																		
F31A	0.0360	**0.1198**	**0.1161**	**0.0930**	**0.0964**	**0.0819**	**0.1248**	**0.0801**	**0.0549**																	
FINO	0.0344	**0.0548**	**0.0494**	0.0214	0.0250	0.0280	**0.0435**	0.0097	0.0353	0.0353																
G14A	**0.1139**	**0.0955**	**0.0694**	0.0091	**0.0149**	**0.0734**	**0.0793**	**0.0407**	**0.1158**	**0.1278**	**0.0515**															
HARW	**0.1340**	0.0004	0.0000	**0.0853**	**0.0834**	0.0021	0.0127	**0.0421**	**0.0661**	**0.1028**	**0.0519**	**0.0749**														
HELG	**0.1255**	0.0075	0.0000	**0.0720**	**0.0775**	0.0000	0.0000	0.0289	**0.0508**	**0.0981**	0.0359	**0.0718**	0.0048													
HORN	**0.0472**	**0.0948**	**0.0984**	**0.0690**	**0.0736**	**0.0820**	**0.1106**	**0.0297**	**0.0358**	**0.0639**	0.0284	**0.0969**	**0.1083**	0.0911												
K10B	**0.1174**	0.0131	0.0099	**0.0685**	**0.0689**	0.0004	0.0113	**0.0410**	**0.0498**	**0.0811**	0.0299	**0.0696**	0.0083	**0.0064**	**0.0924**											
K9A	**0.1079**	**0.0923**	**0.0838**	**0.0208**	**0.0197**	**0.0679**	**0.0892**	**0.0383**	**0.1170**	**0.1248**	**0.0466**	**0.0220**	**0.0822**	0.0846	**0.0817**	**0.0730**										
L10G	**0.0975**	**0.1001**	**0.0800**	0.0090	0.0059	**0.0755**	**0.0822**	**0.0333**	**0.1147**	**0.1216**	**0.0337**	0.0056	**0.0788**	**0.0747**	**0.0862**	**0.0676**	0.0102									
L15A	**0.1230**	**0.1094**	**0.0929**	**0.0447**	**0.0483**	**0.0876**	**0.1068**	**0.0553**	**0.1341**	**0.1491**	**0.0748**	**0.0348**	**0.0874**	**0.0964**	**0.1047**	**0.0975**	0.0132	**0.0345**								
LIMF	**0.0721**	0.0194	**0.0207**	**0.0594**	**0.0607**	0.0141	**0.0333**	0.0095	0.0218	**0.0771**	0.0123	**0.0686**	**0.0332**	**0.0222**	**0.0275**	**0.0319**	**0.0581**	**0.0597**	**0.0717**							
LISB	**0.1256**	0.0511	**0.0780**	**0.1495**	**0.1522**	**0.0642**	**0.0911**	**0.0830**	**0.0569**	**0.1151**	**0.0757**	**0.1656**	**0.0780**	**0.0860**	**0.0742**	**0.0778**	**0.1322**	**0.1459**	**0.1454**	**0.0399**						
PAAL	**0.0689**	**0.0455**	**0.0447**	**0.0390**	**0.0413**	**0.0320**	**0.0602**	0.0115	**0.0455**	**0.1010**	0.0158	**0.0510**	**0.0600**	**0.0501**	**0.0222**	**0.0533**	**0.0378**	**0.0454**	**0.0550**	0.0000	**0.0628**					
Q13A	**0.0896**	**0.0886**	**0.0756**	0.0045	0.0095	**0.0627**	**0.0882**	**0.0305**	**0.1134**	**0.1010**	**0.0380**	0.0090	**0.0703**	**0.0738**	**0.0729**	**0.0667**	**0.0116**	0.0089	0.0273	**0.0602**	**0.1309**	**0.0384**				
SCHV	**0.0768**	0.0120	**0.0164**	**0.0534**	**0.0588**	0.0027	**0.0299**	0.0171	**0.0320**	**0.0764**	0.0189	**0.0609**	**0.0279**	**0.0188**	**0.0438**	**0.0226**	**0.0557**	**0.0638**	**0.0717**	0.0052	**0.0498**	0.0094	**0.0497**			
SYLT	**0.0572**	**0.0956**	**0.1035**	**0.0603**	**0.0721**	**0.0803**	**0.0945**	**0.0377**	**0.0472**	**0.0573**	0.0039	**0.1069**	**0.1069**	**0.0772**	**0.0294**	**0.0777**	**0.0895**	**0.0778**	**0.1211**	**0.0400**	**0.0890**	**0.0497**	**0.0892**	**0.0594**		
WZA	**0.0956**	**0.0837**	**0.0648**	0.0000	0.0034	**0.0635**	**0.0761**	**0.0232**	**0.1031**	**0.1204**	**0.0319**	0.0007	**0.0722**	**0.0676**	**0.0720**	**0.0646**	**0.0132**	0.0011	**0.0278**	**0.0476**	**0.1377**	**0.0269**	0.0001	**0.0452**	**0.0766**	
WZB	**0.1273**	**0.0943**	**0.0747**	0.0040	0.0079	**0.0693**	**0.0820**	**0.0500**	**0.1313**	**0.1349**	**0.0520**	0.0052	**0.0748**	**0.0714**	**0.1085**	**0.0665**	**0.0208**	0.0079	**0.0415**	**0.0750**	**0.1653**	**0.0561**	0.0062	**0.0633**	**0.1053**	0.0037

Table S2. Migration rates.

Number of effective migrants (2Nm) between pairs of samples, estimated using coalescent simulations. Left column holds locations of origin, top row holds locations of destination. For location abbreviations see table 1.

	BG1x	BKUM	BLYT	BRES	BVW	CALA	D15A	EUR7	EURW	F31A	FINO	G14A	HARW	HELG	HORN	K10B	K9A	L10G	L15A	PAAL	Q13A	SCHV	SYLT	WZA	WZB
BG1x		0.3748	15.37	12.62	12.62	12.62	5.772	5.872	1.774	0.1249	8.821	11.82	6.472	16.07	4.823	14.42	3.673	1.93	3.523	3.273	7.221	12.32	5.872	6.422	9.12
BKUM	1.949		4.823	7.621	18.07	18.67	5.972	6.222	5.056	1.724	25.06	8.771	21.76	8.071	5.625	9.07	0.3748	4.573	0.8306	14.72	11.82	23.56	7.821	6.972	9.27
BLYT	4.436	3.973		11.37	16.52	13.77	16.57	10.57	4.873	2.092	22.56	12.62	19.07	19.37	9.52	19.97	0.2179	10.02	1.71	13.62	5.372	12.82	16.42	13.82	10.82
BRES	3.11	0.02499	4.373		25.81	16.17	9.77	9.47	3.201	2.106	29.86	21.66	17.22	31.61	6.446	24.41	1.439	6.972	2.003	13.07	15.57	18.37	12.27	16.12	12.57
BVW	3.187	8.771	12.57	25.36		24.01	11.62	10.82	5.675	2.179	29.56	11.07	26.21	34.71	10.42	24.26	1.7	4.287	1.061	22.41	10.27	26.01	14.02	15.07	16.12
CALA	4.273	8.771	7.721	30.16	26.61		12.92	7.571	6.022	2.46	27.91	9.77	24.21	30.41	10.52	24.36	0.9615	7.114	1.97	13.77	12.67	27.36	14.17	16.72	13.82
D15A	3.294	3.423	10.42	11.27	9.52	6.622		7.771	4.009	1.906	24.41	10.12	11.12	28.01	5.655	7.663	0.9922	6.153	1.95	18.32	8.521	6.372	10.67	7.471	6.072
EUR7	3.522	9.37	8.221	17.17	16.67	0.7746	9.12		0.1634	2.062	18.37	8.321	16.87	12.02	10.62	9.32	1.589	5.264	1.39	18.97	3.473	15.07	10.97	7.071	5.522
EURW	2.926	8.421	9.82	10.17	10.02	13.72	9.12	0.2249		2.443	12.37	8.321	20.56	10.92	6.772	22.71	2.348	7.471	1.524	12.87	6.772	29.71	4.923	8.771	7.871
F31A	0.02499	0.3248	1.274	13.62	1.374	14.62	3.723	12.07	5.372		6.422	13.17	8.171	12.72	6.072	12.17	7.921	2.674	2.174	2.124	5.372	14.37	6.822	7.921	7.321
FINO	2.899	8.421	10.72	27.61	17.52	13.07	19.52	8.121	3.716	2.224		15.92	15.07	17.17	5.327	21.36	11.97	0.4003	4.273	16.07	11.82	22.21	13.07	19.52	8.521
G14A	3.367	1.124	9.32	28.06	9.87	9.32	13.07	7.346	4.045	2.215	27.66		10.52	34.11	2.237	16.42	1.511	6.951	2.267	10.22	8.321	13.67	9.47	17.37	16.72
HARW	2.519	9.97	9.57	20.01	24.91	12.77	12.22	8.071	4.294	2.072	20.01	8.671		25.46	9.87	21.81	2.338	6.222	2.501	18.37	14.52	30.26	9.32	9.12	11.32
HELG	4.959	4.773	14.47	37.16	30.06	22.21	24.11	8.771	5.326	2.305	24.96	27.56	20.36		10.27	19.77	1.206	9.62	2.414	17.97	17.57	23.71	9.62	15.47	4.173
HORN	3.573	0.02499	11.97	15.07	17.72	2.024	8.921	9.97	5.27	2.665	16.32	7.989	13.92	23.56		15.77	2.503	8.521	1.632	16.37	10.92	22.06	20.46	1.924	9.02
K10B	3.837	0.1749	7.621	26.96	24.56	19.37	8.771	9.42	5.164	2.481	26.91	11.02	19.92	21.46	7.738		1.473	8.259	1.067	13.72	20.06	17.72	13.12	18.97	12.42
K9A	2.582	0.5747	0.007363	1.978	2.409	0.123	0.6377	0.8244	2.236	2.23	28.61	1.258	1.204	2.746	1.063	1.281		0.3957	4.052	3.094	0.377	1.125	22.31	1.039	5.472
L10G	1.269	0.02499	11.27	11.47	8.471	8.121	10.42	6.061	4.973	1.769	23.36	16.22	9.47	23.11	6.122	16.02	0.7773		1.526	25.41	7.521	6.722	19.47	5.672	6.122
L15A	2.646	0.02121	0.01156	0.7772	0.3425	0.01539	0.8689	0.8792	1.296	2.074	9.57	0.3128	0.02011	1.766	0.5227	0.03981	13.22	0.152		0.01854	0.01306	0.1698	8.071	0.2249	5.772
PAAL	3.095	20.96	4.423	17.77	26.01	16.57	17.32	10.07	5.333	0.02499	26.91	16.47	22.56	26.26	0.02499	21.76	2.644	13.82	2.397		9.77	28.61	11.67	10.47	11.02
Q13A	3.823	5.572	4.523	29.06	23.46	10.27	7.371	6.422	5.067	2.524	22.66	13.27	18.87	23.61	5.922	24.46	1.348	6.808	1.516	15.42		14.37	9.37	16.07	13.12
SCHV	4.102	16.22	10.97	33.41	23.61	21.71	11.22	8.471	6.272	2.524	23.76	14.72	27.86	28.21	10.89	19.07	3.153	6.085	2.733	17.17	17.62		13.12	18.97	16.52
SYLT	2.631	8.321	8.321	27.56	17.62	10.12	11.27	4.573	4.021	1.937	21.36	12.72	11.52	20.51	7.921	19.37	15.37	6.465	4.523	5.372	1.924	20.71		16.67	7.671
WZA	2.883	0.02499	7.621	21.81	20.06	15.57	7.671	1.924	4.365	1.914	24.91	17.92	13.67	27.81	6.823	18.12	1.574	6.247	2.458	11.82	14.07	17.07	13.97		10.32
WZB	2.875	11.52	7.771	33.16	29.61	14.12	10.57	7.121	4.445	2.024	25.51	21.21	16.02	22.96	7.221	17.47	5.772	6.17	3.803	14.87	14.37	23.51	9.62	11.12	

Table S3. Particle matrix.

Total number of arriving particles between sampling locations, resulting from DELFT3D-FLOW and DELFT3D-PART model, the latter simulating larval dispersal for 70 days. Left column holds locations of origin, top row holds locations of destination. For location abbreviations see Table 1.

	BG1x	BKUM	BLYT	BRES	BVW	CALA	D15A	EUR7	EURW	F31A	FINO	G14A	HARW	HELG	HORN	K10B	K9A	L10G	L15A	PAAL	Q13A	SCHV	SYLT	WZA	WZB
BG1x	29.62838	0	0	0	0	0	0	0	0	1.649977	0.002562	0.340478	0	0	0	0	0	0	0	0	0	0	0	0	0
BKUM	0	115.563	0	0	0	0	0	0	0	0	0.027224	0	0	2.989115	0.005909	0	0	0	0	0	0	0	0	0	0
BLYT	0	0	47.1927	0	0	0	0	0	0	0	0	0	0	0	0	0	0	0	0	0	0	0	0	0	0
BRES	0	0.000577	0	1167.466	0	0	0	1.466777	0	0	0	0.003446	0	0	0	0	2.66E-05	0.000983	0.054394	0.06104	0.569421	0.37573	0	0	0
BVW	0	0.20374	0	0	11.30357	0	0	0.002121	0	0	0	0.658894	0	0	0	0	0.000195	0.041345	0.531428	0.019687	0	0	0	0.007852	0.012197
CALA	0	0	0	0.282479	4.34E-05	543.8507	0	0.602714	0.089809	0	0	0	0	0	0	0	0	0	0	0	0.033504	0.000716	0	0	0
D15A	0.000353	0	0	0	0	0	31.08992	0	0	0.673546	0	0	0	0	0	0	0	0	0	0	0	0	0	0	0
EUR7	0	0.010597	0	0.164612	3.68E-06	0	0	6.53168	3.30E-06	0	0	0.091257	0	0	0	0	0	0.001337	2.032783	1.904748	8.834629	3.293846	0	0.000123	0.001564
EURW	0	0	0	0	6.420489	0	0	2.138403	18.24299	0	0	0.030467	0	0	0	0	0.003811	0.065449	0.948309	0.012709	0.005701	0.000292	0	0	0
F31A	0	0	0	0	0	0	0	0	0	31.93388	0	0	0	0	0	0	0	0	0	0	0	0	0	0	0
FINO	0	0	0	0	0	0	0	0	0	0	35.07171	0	0	0	0	0	0	0	0	0	0	0	0	0	0
G14A	0	0	0	0	0	0	0	0	0	0	0.590233	38.30031	0	0	0.000374	0	0	0	0	0	0	0	0	0	0
HARW	0	0	0	0	0	0	0	0	5.61E-05	0	0	0	1102.512	0	0	0	0	0	0	0	0	0	0	0	0
HELG	0	0	0	0	0	0	0	0	0	0	0.538469	0	0	30.97328	1.498179	0	0	0	0	0	0	0	0.070195	0	0
HORN	0	0	0	0	0	0	0	0	0	0	0	0	0	0	65.68903	0	0	0	0	0	0	0	0	0	0
K10B	0	0.001958	0	0	0	0	0	0	0	0.003137	0.002705	0.270754	0	0	0	12.28087	0.584606	0.694237	0	0	0	0	0	0	0
K9A	0	0	0	0	0	0	0	0	0	0.002602	0.088494	1.399617	0	0.000655	0	0	40.06836	4.961225	0	0	0	0	0	0	0
L10G	0	7.90E-06	0	0	0	0	0	0	0	0.000177	0.142311	0.926901	0	0.002073	0	0	1.516391	39.32079	0	0	0	0	0	0	0
L15A	0	4.377627	0	0	0	0	0	0	0	0	0.043257	0.037732	0	0.14377	0	0	0	0	204.74	0.000738	0	0	0	3.204995	2.233253
PAAL	0	2.926629	0	0	0	0	0	0	0	0	0.021107	0.085842	0	0.041937	0	0	0	2.87E-05	4.937252	631.6918	0	0	0	1.466177	0.905502
Q13A	0	0.447708	0	0	0	0	0	8.22E-06	0	0	8.66E-05	0.280723	0	0	0	0	0	0.013703	3.74662	4.530116	12.72411	4.502403	0	0.042796	0.061654
SCHV	0	0.869073	0	0	0	0	0	0.000159	0	0	0.000221	0.210189	0	4.14E-05	0	0	0	0.00262	3.964979	5.691386	7.610964	20.78731	0	0.116239	0.119129
SYLT	0	0	0	0	0	0	0	0	0	0	0.031264	0	0	0	1.372142	0	0	0	0	0	0	0	278.2046	0	0
WZA	0	1.517607	0	0	0	0	0	0	0	0	0.000239	0	0	0.077578	0.000133	0	0	0	0	0	0	0	0	2573.495	884.2185
WZB	0	1.740847	0	0	0	0	0	0	0	0	0	0	0	0.00558	0	0	0	0	0	0	0	0	0	1920.336	1788.836

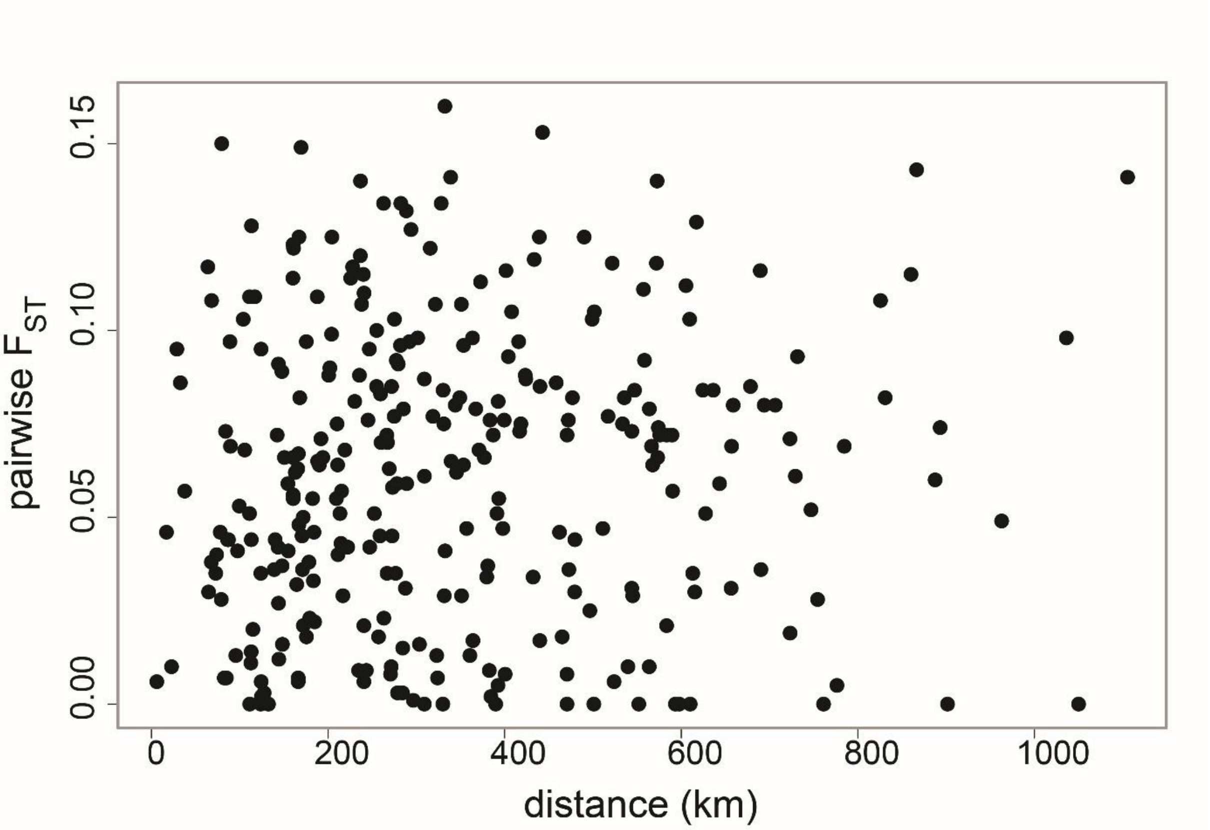

Figure S4: No isolation by distance in *Mytilus edulis* in the southern North Sea. Plot of linear geographical distance among 25 sampling locations against pairwise F_{ST} based on microsatellite data, showing no correlation (Mantel test, r=0.078, p=0.22)

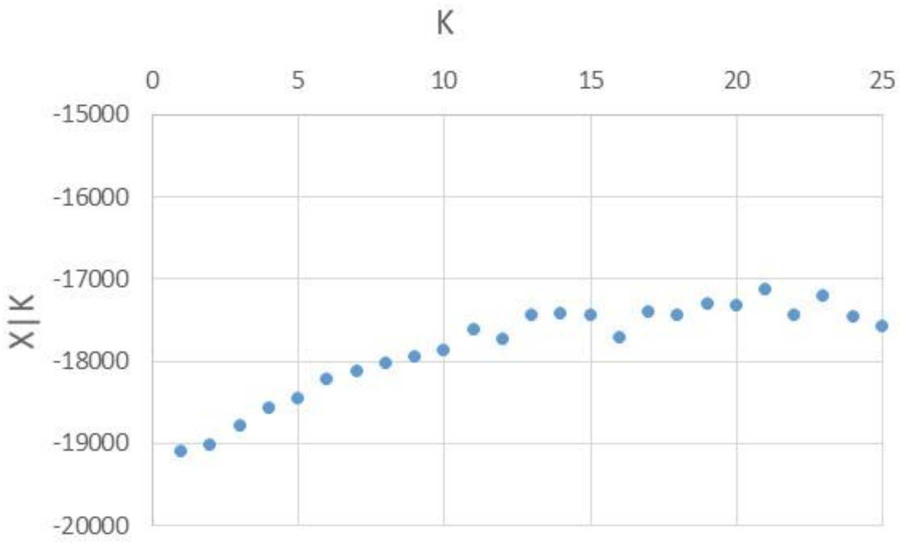

Figure S5: Structure results for *M. edulis* in the southern North Sea.
Relationship between likelihood of the data given number of groups (X|K) and number of groups (K) as estimated using the Bayesian clustering software Structure.

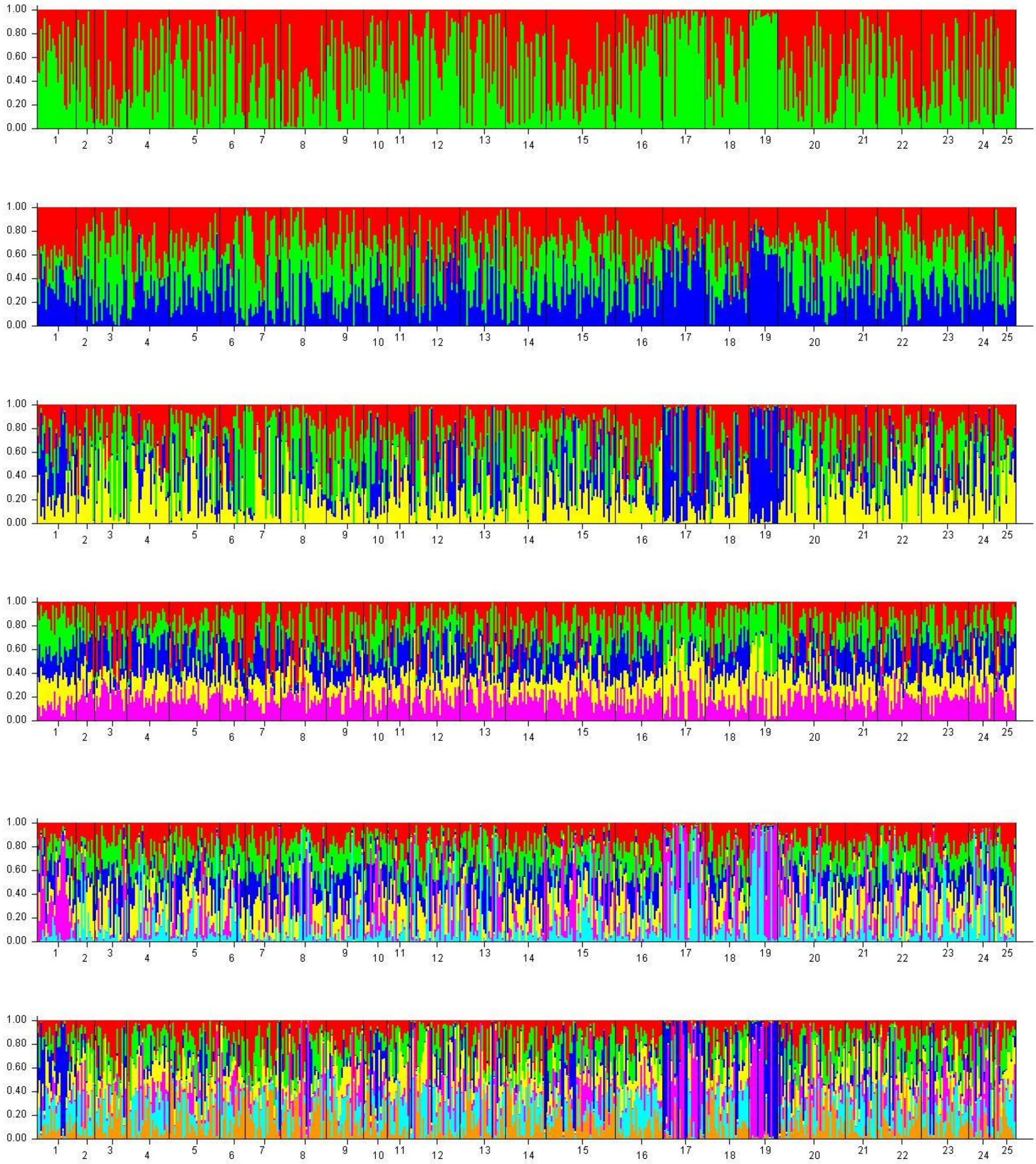

Figure S6: Structure barplot results for *M. edulis* in the southern North Sea. Runs consisted of 100,000 burn-in steps followed by 100,000 repeats. From top to bottom: K = 2 through 7. In each barplot, every vertical coloured bar indicates genetic composition of an individual according to K groups. Groups of individuals are coded 1-25 corresponding in alphabetical order to samples BG1x through WZB (see Table 4).

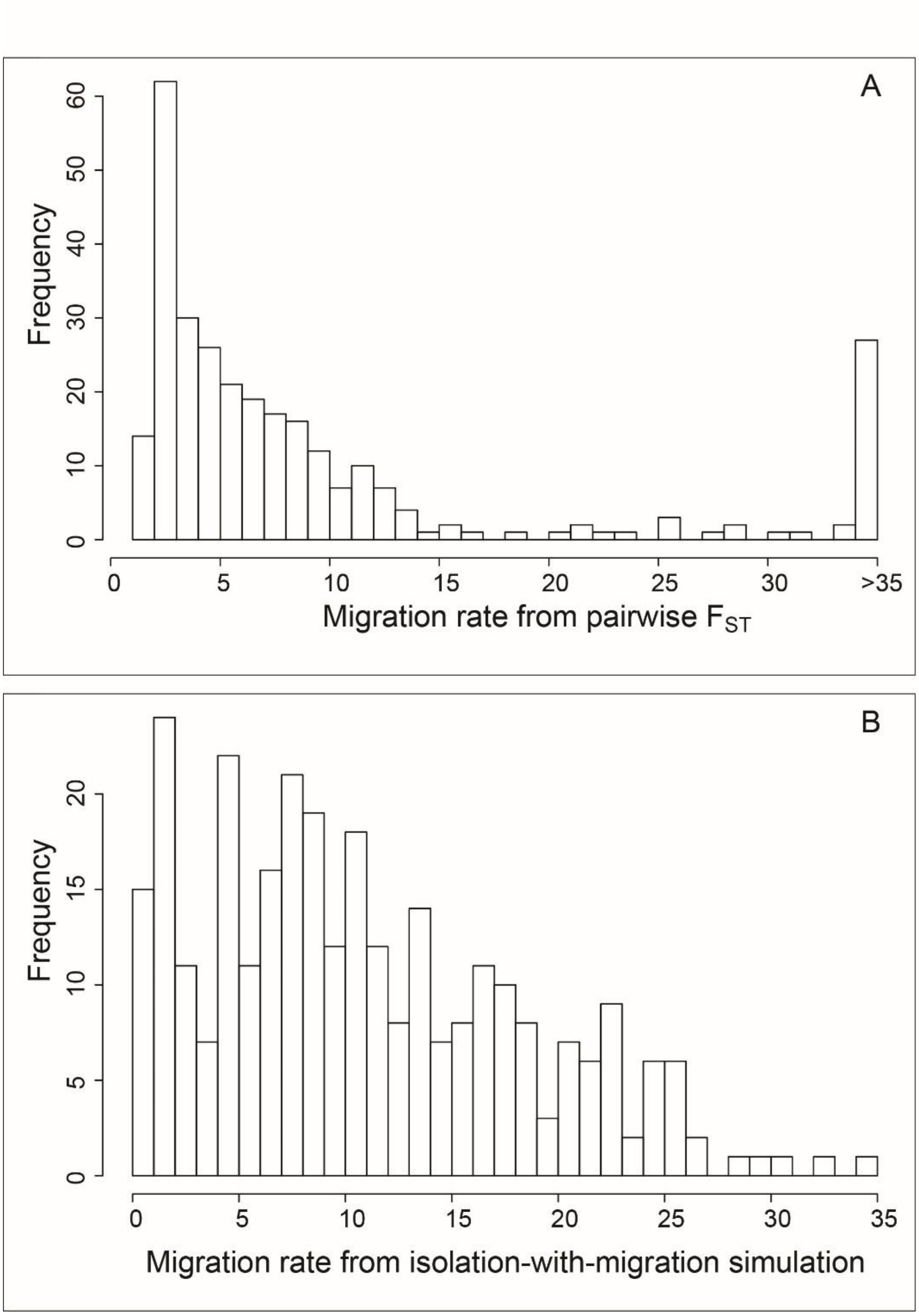

Figure S7: Distributions of estimated migration rates.

Distribution of migration rates estimated on the basis of pairwise F_{ST} values, converted to 2Nem's following Wright's infinite island model (A), and on the basis of coalescent isolation-with-migration simulations (average between bidirectional migration rates (B).
